# Stakeholder perspectives on depression management: A design thinking exploration for person-centered digital health

**DOI:** 10.1371/journal.pone.0341431

**Published:** 2026-02-09

**Authors:** Sònia Moretó, Aïna Fuster-Casanovas, Andrea Duarte, Noemí Robles, Daniela Cabutto, Antoni Pérez-Navarro, Irene Lapuente, Emma Planas, Maria Padilla, Carme Carrion

**Affiliations:** 1 eHealth Lab Research Group, School of Health Sciences and eHealth Centre, Barcelona, Spain,; 2 Unitat d’Innovació, Direcció de Qualitat, Processos i Innovació, Hospital Universitari Vall d’Hebron, Barcelona, Spain,; 3 Canary Islands Health Research Institute Foundation (FIISC), El Rosario, Spain,; 4 Network for Research on Chronicity, Primary Care, and Health Promotion (RICAPPS), Barcelona, Spain,; 5 Evaluation Unit (SESCS), Canary Islands Health Service (SCS), El Rosario, Spain,; 6 Data Science Lab, UOC Labs, Universitat Oberta de Catalunya, Barcelona, Spain,; 7 Faculty of Computer Science, Multimedia and Telecommunication, Universitat Oberta de Catalunya (UOC), Barcelona, Spain,; 8 Escola Universitària Salesiana de Sarrià (EUSS), Barcelona, Spain,; 9 La Mandarina de Newton, Barcelona, Spain,; 10 Research and Innovation Unit, Costa del Sol University Hospital, Marbella, Spain,; 11 School of Medicine, Universitat de Girona (UdG), Girona, Spain; School of Nursing Sao Joao de Deus, Evora University, PORTUGAL

## Abstract

**Introduction:**

eHealth has the potential for managing depression and enhancing quality of life. Identifying end user needs and employing participatory methodologies that actively engage all stakeholders can improve user experience, usability and effectiveness.

**Materials and Methods:**

Six face-to-face empathy workshops were conducted in three Spanish autonomous communities (Catalonia, Andalusia and Canary Islands) using design thinking methodologies, involving individuals with depression and mental health professionals. Data were analyzed using an iterative and inductive analysis approach.

**Objective:**

To explore the perspectives of people diagnosed with depression and healthcare professionals involved in its management, using a design thinking methodology.

**Results:**

Thirteen individuals with depression (10 women, average age 49.15, SD: 18.10) and 17 mental health professionals (11 women, average age 40.21, SD: 12.15) participated in empathy workshops. Three key themes emerged: the daily experience of depression, the potential of technology in managing depression, and emerging challenges to address.

**Discussion:**

The intensity and daily experience of depressive episodes were influenced by various factors. Technologies, when used as a complement to face-to-face care, showed potential for managing depression. However, there were associated risks and challenges that need to be addressed.

**Conclusion:**

It is essential to identify and understand the needs of end users and incorporate the perspectives of all stakeholders in the design and development of digital health interventions.

## Introduction

Approximately 280 million people worldwide are diagnosed with depression, representing a prevalence of 5% among the adult population and 5.7% among people over 60 years of age [1]. The World Health Organization (WHO) has predicted that depression will become the leading cause of disease burden by 2030, highlighting the urgent need for comprehensive and strategic plans to address its management [2]. Findings from the WHO Mental Health Survey revealed that only one in 5 people in high-income countries and just one in 27 people in low-income countries receive adequate treatment for depression [3].

Current clinical practice guidelines recommend a stepped care model, in which treatment is tailored to the severity of symptoms, previous treatment experiences and individual preferences. This model emphasizes the importance of person-centered care, with shared decision making and personal empowerment as key strategies [4]. In real practice, this translates into a combination of different pharmacological and non-pharmacological treatments, including both individual and group cognitive-behavioural therapy [4–6].

In recent years, the use of digital health interventions for depression has gained attention, showing promising results. For instance, Stuart et al. [7] compared internet-based cognitive behavioural therapy (iCBT) with standard treatment for depression, finding that iCBT significantly improved depressive symptoms and remission rates at 8 weeks. Similarly, Hungerbuehler et al. [8] evaluated the effectiveness of domiciliary treatment for mild depression using psychiatric video consultations compared to face-to-face consultations. The results showed a significant reduction in depressive symptomatology at 12 months in both groups; thus, it was shown that psychiatric treatment through video consultations could be considered feasible and as effective as face-to-face therapy.

Other solutions, such as mobile health (mHealth) have also emerged as valuable tools. A systematic review and meta-analysis found that mHealth interventions significantly reduced depressive symptoms compared to minimal interventions, with hybrid approaches (combining digital tools with in-person care) proving to be the most effective [9]. Another recent systematic review and meta-analysis of digital interventions in primary care further confirmed their effectiveness, particularly when multiple tools were integrated, as opposed to using a single tool alone [10].

These findings highlight the potential of digital health solutions to improve depression care with the potential to improve people’s quality of life. However, successful implementation in clinical settings requires a comprehensive, person-centered approach that actively involves all stakeholders, including patients and healthcare professionals. As proposed by The National Institute for Health and Care Excellence (NICE) [4], shared decision-making is critical to ensure that interventions are aligned with individual values, preferences and lived experiences. Many individuals may not identify with or respond to traditional pharmacological treatments, making it essential to incorporate their perspectives in designing new care pathways.

One approach that supports this goal is design thinking, a person-centered, iterative methodology that fosters empathy, creativity, and collaboration in solving complex problems. It supports the idea that not all problems need to be solved through a rational approach, but design thinking is a rather more viable approach to address complex problems where there is often ambiguity between possible solutions [11–14]. Originally developed at the Stanford d.school, the design thinking model is an iterative and interactive process structured in five interconnected stages: (1) empathization, (2) definition, (3) ideation, (4) prototyping, and (5) validation [15–17].

In the empathization phase, the objective is to understand what truly matters to the stakeholders involved in the problem that needs to be addressed. The definition phase involves synthetizing information from the previous phase to identify key insights and challenges from the perceptions and needs of the stakeholders. While these first two phases focus on understanding the needs and discovering areas for improvement, the following phases are focused on co-creating, testing and validating solutions based on the empathization and definition phases. Thus, the main objective of ideation is that stakeholders envision solutions to the identified problems. In ideation, stakeholders collaboratively generate ideas to address the challenges identified. Prototyping allows them to give form to their ideas, which are then presented and iteratively improved. Finally, the validation phase ensures that the proposed solutions align with stakeholders’ expectations and needs [17].

While many eHealth initiatives have been shown to be effective in the management of depression, their success ultimately depends on how well they reflect the real needs, values and experiences of stakeholders. Empathy, co-creation and active participation of both patients and professionals are essential components of person-centered digital health design. This highlights the need to adopt a person-centered care model that not only considers clinical outcomes but truly values people’s lived experiences, personal

goals, and individual preferences [18]. In depression care this is even more crucial, as it acknowledges the complexity and subjectivity of each person’s experience with the condition [19]. A person-centered approach prioritizes hearing and understanding what is most important to patients in their everyday lives, such as emotional, social, and contextual aspects that lie beyond conventional biomedical frameworks. Applied to digital health, this viewpoint supports the imperative to create technologies that are not just clinically valid, but also emotionally engaging, usable, and aligned to the user’s identity and lifestyle. Evidence shows that person-centered approaches have the potential to improve depression outcomes. They require individualized and participatory planning based on personal needs and shared decision-making. It is noteworthy to acknowledge that in some cases individuals may face challenges in fully engaging with these approaches [19]. However, an exploratory mapping review of digital mental health interventions revealed that, although some efforts have been made to incorporate human-centered design principles, these often lack the involvement of trained designers and design research methodologies, limiting their ability to fully address user needs and preferences [20]. By integrating design expertise and a person-centered model, digital interventions can become more responsive, engaging, and empowering, fostering greater adherence, satisfaction, and ultimately, better mental health outcomes.

This study aims to explore the perspectives of people diagnosed with depression and healthcare professionals involved in its management, using design thinking methodology, specifically through the emphatization phase. By identifying stakeholders’ needs and opportunities for innovation, the study seeks to define future directions of digital health interventions that are both effective and truly person-centered.

## Methods

### Study design

Using design thinking, a human-centered design methodology, six face-to-face empathy workshops (three with people diagnosed with depression and three with mental health professionals) were conducted across three autonomous communities in Spain: Catalonia, Andalusia and the Canary Islands. The main objectives of these workshops were three: to understand the daily life of depressive episodes, explore the potential opportunities offered by technology from the perspective of different stakeholders, and define new challenges to be explored according to their needs.

### Study participants

Participants in the empathy workshops for healthcare professionals included those actively involved in depression management, recruited between 1 October 2023 and 20 February 2024. Members of the research team, affiliated with different healthcare institutions, completed the recruitment procedure following the snowball method [21] but they did not engage in the workshops as participants.

For empathy workshops targeting people diagnosed with depression, people with a current diagnosis of depression or who had received a diagnosis within the past two years were invited to participate. Recruitment occurred between October 2023 and February 2024, using two main strategies. Initially, as with healthcare professionals, the snowball method was used by recruiting people currently receiving care from participating professionals [22]. The second strategy involved indirect recruitment through informational posters displayed in community pharmacies, primary care centers and hospitals, as well as digital dissemination of the project through the social channels managed by the Universitat Oberta de Catalunya (UOC), Participa y Decide sobre tu Salud (PyDeSalud) and the Evaluation Unit of the Canary Islands Health Service (SESCS). In both posters and digital dissemination materials, the contact information of a member of the research group was provided. Participants were able to contact the researcher via email to verify whether they were eligible for inclusion, obtain the necessary information about the study, solve any doubts, and make a decision about participating in the workshops.

### Study setting

In Catalonia, an empathy workshop for people diagnosed with depression were hosted at the UOC headquarters. The workshop for professionals took place at the Universitat de Girona. In Andalusia, an empathy workshop for people with depression were conducted at the Primary Care Health District Costa del Sol. For professionals, one workshop was held at the Primary Care Health District Costa del Sol. Finally, both workshops in the Canary Islands were held at the headquarters of the Directorate of the Canary Islands Health Service.

### Empathization sessions

Workshops occurred between November 2023 and February 2024 and were designed and facilitated by an expert in design thinking methodology and its application in mental health. Each session lasted approximately two hours, and data collection occurred through detailed written documentation: the materials generated by participants during the workshops, as well as the notes taken by the moderator. Two research team members attended each session as observers.

Each workshop consisted of four structured dynamics:

- **Warm-up:** The warm-up is an activity to smooth the interaction between participants with individual presentations. Participants introduced themselves through images, one representing their identity and another their relationship with technology. Participants could use a chosen pseudonym to protect their privacy.
- **Mind Map:** A mind map is a diagram that helps to visualize different related concepts that branch out from a key concept placed in the center [23]. For people with depression, the key concept was “themselves”, and the goal of the dynamic was to identify those concepts that were considered important in their daily lives. For professionals, the key concept was again “themselves” and the objective was to identify those factors that they consider to their clinical approach to depression. Each participant received a DIN-A3 sheet to complete the mind map individually, followed by a group-sharing discussion.
- **Customer Journey:** The customer journey is a technique to evaluate how customers interact with an organization, and therefore it can be extrapolated to the health field [24,25]. For both professionals and people with depression, this activity aimed to identify positive and negative factors affecting participants’ relationship with their environment and seasonal variations. Participants completed a DIN-A3-sized chronological template mapping experience across months. After completing the activity, participants were invited to share their customer journey with the rest of the group.
- **Storyboard:** The storyboard is a visual tool used to graphically tell a person’s story from the user’s point of view [26,27]. Divided into small groups, participants employed this technique to visualize their personal stories, highlighting how technology could improve their experiences. Groups used DIN-A4 templates with six predefined panels in which participants had to represent how they imagined technology could help them in their daily life. After completion, each group presented their storyboard to fellow participants.

At the end of each session, participants were thanked and informed about subsequent steps, including receiving an analyzed results report. Additionally, they were also asked for their willingness to participate in future research, with contact details collected from those interested.

### Data analysis

Three researchers (SM, AFC and IL) analyzed the written materials generated across all workshops using reflexive thematic analysis with an iterative and inductive approach. First, they familiarized themselves with the corpus and wrote reflexive memos. Each analyst then performed open coding on an initial subset of the data independently, allowing codes to emerge rather than applying pre-existing categories. The code set was then refined across successive cycles. They clustered and renamed codes, developed patterns and defined overarching themes. Any interpretive differences were resolved through discussion and consensus. After developing themes inductively, they organized the synthesis against the study objectives.

### Ethics

The EvalDepApps project has the approval of the University Institute for Primary Care Research (IDIAP) Jordi Gol Health Care Ethics Committee (code 23/051-P). Information about participating in the project was provided to all those who expressed an interest, as well as the e-mail address of a team member to answer any questions that might have arisen. Once participants understood the terms of participation, they signed a written informed consent form before the workshops began.

## Results

### Participants

A total of 13 people from the three autonomous communities participated in the empathization workshops for people diagnosed with depression (5 in Catalonia, 7 in Andalusia and one in Canary Islands). Across all groups, 10 were women, with a mean age of 49.15 years (SD: 18.10). Participants reported additional conditions alongside depression including fibromyalgia, cancer, anxiety, eating disorders, and other psychological disorders.

On the other hand, a total of 17 people from the three autonomous communities participated in the empathy workshops for health professionals 8 in Catalonia, 5 in Andalusia and 4 in Canary Islands). Across all groups, 11 were women, with a mean age of 40.21 years (SD: 12.50). Participants represented a range of disciplines, including psychiatry, clinical and general health psychology, social work, mental health nursing, general nursing and family and community medicine.

### Daily experience of depressive episodes

The results for the daily experience of depressive episodes were obtained through the mind map and customer journey dynamics.

### People diagnosed with depression

**Fig 1:**
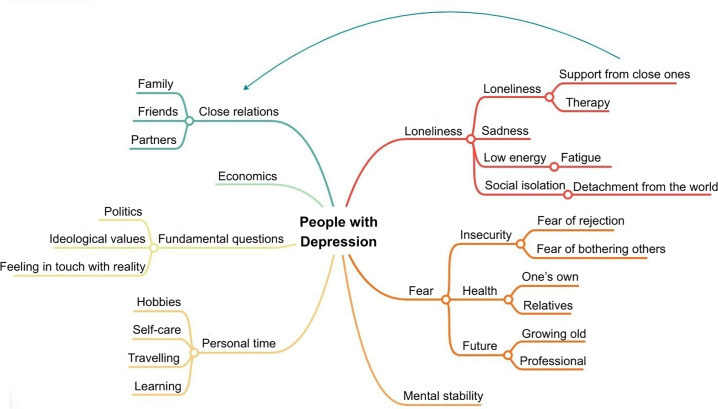
Common keywords identified in the mind map of people diagnosed with depression. This mind map illustrates the most frequently mentioned terms by people diagnosed with depression and the relationships between these terms.

The pattern of keywords identified among people with depression is illustrated in Fig 1. Participants highlighted both positive aspects and barriers that they experience on a daily basis.

Three main positive aspects that contribute to the daily life of people with depression were identified. First, support from close social circles was seen as essential for sharing experiences and needs. Second, following structured routines helped participants achieve goals and maintain autonomy. Finally, engaging in certain artistic activities (e.g., music, dance, painting and photography), travelling, and being in contact with nature were described as sources of well-being in their daily lives.

Regarding barriers, concerns were reported to dominate participants’ thoughts. Crisis moments, such as dissociative episodes, anxiety attacks, and extreme fatigue, were particularly challenging. These crises were often triggered by unwanted life changes (e.g., job changes, family separation) or comorbid conditions (e.g., chronic pain, fibromyalgia, insomnia). Such events led to emotional destabilization, which in turn affected their social, family and professional interactions. Participants noted that these emotional states sometimes led to the adoption of maladaptive coping mechanisms, including substance abuse or excessive use of video games.

### Mental health professionals

**Fig 2:**
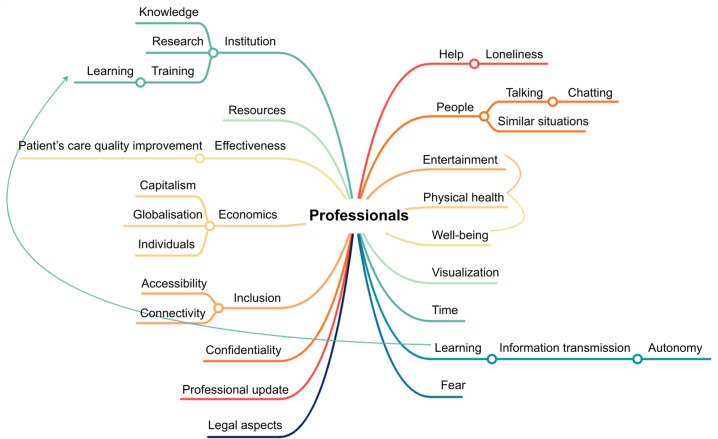
Common keywords identified in the mind map of mental health professionals. This mind map illustrates the most frequently mentioned terms by mental health professionals and the relationships between these terms.

Fig 2 illustrates the keyword patterns identified by health professionals, reflecting their concerns and perceived needs for depression treatment.

Concerns were grouped into two areas. First, participants pointed to institutional culture, resource limitations, and their impact on the quality of mental health care. Legal concerns, such as confidentiality in digital interventions, were also mentioned. Second, professionals highlighted the importance of understanding patients’ socio-economic contexts and the correct classification of depression’s etiology for effective treatment.

Key treatment needs identified included environmental support, opportunities for socialization, establishment and maintenance of routines, physical activity, and activities promoting personal autonomy. On the other hand, a seasonal pattern was also observed: January was seen as a critical month for initiating treatment, while summer holidays often disrupted ongoing support, making it less favorable time to start interventions.

### Opportunities that technology can offer in depression

The results for the opportunities that technology can offer in depression were obtained from the warm-up and story board dynamics.

### People diagnosed with depression

Participants identified and designed several opportunities for technology in depression management. They saw technology as a tool for learning, health promotion, task organization, distraction, and communication. Proposed innovative solutions included a customizable mobile application to support emotional regulation during crisis and a digital tool for sharing information with a health professional to facilitate monitoring and treatment progress. Personalization of technology based on individuals’ needs was emphasized. However, participants also noted that digital solutions should complement not replace professional care.

### Mental health professionals

Professionals viewed digital tools as complementary to in-person care rather than standalone treatments. Opportunities identified included enhancing continuity of care, supporting gradual withdrawal from pharmacological treatment, facilitating follow-up after discharge, and providing ongoing patient support. Digital solutions were proposed to support learning, health promotion, task organization, emotional management, and communication. Innovative examples included customizable mHealth tools for emotional management and for improving monitoring through sharing health information with professionals.

### New challenges to achieve

The results for this objective were obtained from the customer journey dynamic.

### People diagnosed with depression

While participants appreciated the growing availability of digital solutions to improve the management of depression, two major challenges were identified. First, there is a need for guidance and skills to select digital health tools that are safe and effective. In this respect, the scientific evidence supporting a digital health solution was seen as key. Second, participants emphasized the importance of educating users on responsible use of digital tools to prevent dependency or overuse.

### Mental health professionals

Professionals identified the secure implementation of digital health solutions as a primary challenge. From their perspective, training and information are needed to better understand how digital tools can be integrated into the care pathway and their implications for practice. Additionally, professionals noted that digital technologies could facilitate peer communication among people with depression, which might carry risks such as negative reinforcement. To mitigate these risks, supervised forums and/or meetings were recommended, reinforcing the need for a multidisciplinary approach. Other identified challenges included bridging the digital gap and ensuring equitable access to digital health resources.

### Main keywords

A summary was made of the keywords identified in both profiles, as well as in the overall analysis for each objective and dynamic conducted (Table 1).

**Table 1:** Summary of keywords for each objective, dynamic and participant type.

Objective	Dynamic	Common keywords	People diagnosed with depression	Professionals
**Daily life with depressive episodes**	Mind map	People Fear Wellbeing/mental stability	People Economy Questions Personal space and time Loneliness Sadness Lack of energy Isolation Fear Mental stability	Institution Resources Efficiency Economy Inclusion Confidentiality Updates Help People Engagement Exercise Wellbeing Visualization Time Learning Fear
**Opportunity that technology can offer**	Warm up	Cover the needs Coadjutant therapy	Training Distraction Coadjutant therapy	Coadjutant therapy
	Story board	Technology: positive aspect Monitoring/follow up Socializing/ support	Getting back to reality Safe space Personalization Follow up Socializing Communicate Routines	Adherence to treatments Support for stopping pharmacological treatment Follow up Support
**New challenges to achieve**	Customer journey	Difficulty choosing the tool Education Lack of knowledge Responsible use	Difficulty choosing the tool Education Scientific evidence Connection with people	Difficulty choosing the tool Technology contribution Professional implication Negative feedback Control Digital gap Multidisciplinary approach

## Discussion

The objective of this study was to explore the daily experience of depressive episodes, identify the opportunities technology can bring to different stakeholders, and define new challenges based on their needs. The findings highlight several important considerations for addressing the management of depression through digital health solutions.

In the present study, people diagnosed with depression considered that adverse life events, including stressors and personal or professional difficulties, were contributing factors to depressive symptoms. In fact, adverse life events are considered risk factors for depression [28–30]. Furthermore, it is not only the occurrence of such events, but also the individual’s perception of them that influences symptoms progression and coping ability [29,31,32]. For example, a longitudinal study following a cohort of people with dysthymia and depression over 12 years found that those who experienced positive environmental events had significantly fewer symptoms than those exposed to negative events [29].

Regarding comorbidities, the results of this present study suggested a relationship between depression and comorbid conditions such as insomnia, chronic pain and fibromyalgia [33–36]. For instance, Lee et al. [33] suggested a significant comorbidity between depression, insomnia, migraine and fibromyalgia. However, in the study it was observed that the severity of insomnia and fibromyalgia increased with additional comorbidities, whereas this pattern was not observed in depression. These co-occurrences point to the need for an integrated therapeutic approach from a multidimensional perspective. From this perspective, person-centered care and design thinking provide a valuable framework, as it encourages a holistic understanding of people’s physical, emotional, and social needs, and promotes care strategies that align with their lived experiences and personal goals.

Moreover, professionals in this study also noted seasonal variations in depression, indicating January as particularly challenging and March to June as relatively more stable periods. These findings align with known patterns of Seasonal Affective Disorder (SAD) [37,38], and it is possible that it influences the improvement of depressive symptomatology. However, SAD has a specific symptomatology and our study did not assess specific symptomatology across seasons. Future research should explore the impact of seasonal variation on depression and quality of life in greater depth. It is important to note that these patterns may vary depending on the geographical location, as the study was conducted in the Northern Hemisphere, where seasonal variations may have a specific impact.

Participants in both groups identified the potential of technology in addressing depression.. First, people diagnosed with depression acknowledged the value of digital health tools for improving well-being. In this context, the level of health literacy plays a crucial role in health outcomes [39]. Particularly, mental health literacy is associated with reduced stigma, improved mental health, and increased help-seeking behavior [40,41]. A systematic review and meta-analysis by Yeo G et al. [42] showed that digital mental health interventions can improve and maintain health literacy, reduce depression, anxiety, loneliness, and internalizing and externalizing symptoms, while enhancing resilience and quality of life. These findings support participants’ views on the role of digital tools, and suggest that future research should further examine the role of digital mental health interventions in improving mental health literacy and mental health outcomes.

Second, professionals viewed technology as beneficial for monitoring routines and promoting healthy habits, which were also considered essential by people with depression. Prior research has demonstrated associations between routine disruption and mood disorders [43], and several studies support the role of lifestyle interventions in reducing depressive symptoms [44,45]. In fact, a meta-analysis showed a greater effect of lifestyle interventions that combined face-to-face and telephone contact, compared to those interventions given separately [44].

Third, both people diagnosed with depression and professionals emphasized that digital tools should complement, not replace, face-to-face care. People diagnosed with depression highlighted the importance of maintaining direct professional contact, and professionals were especially supportive of hybrid interventions because this type of therapy may offer additional benefits, such as improved continuity of care. This aligns with evidence showing that hybrid interventions are effective in improving both health outcomes, including depressive symptoms and quality of life [9,46,47]. Furthermore, the potential for continuous monitoring in these interventions may also enhance treatment adherence [48], a key issue in depression care that several studies have sought to improve using digital strategies [49–51].

However, several challenges that should be addressed were highlighted by participants. The need for education and training in the use of digital tools for the management of depression was underlined. Existing evidence underlines the importance of training digital competences in different health fields in order to improve wellbeing and health outcomes [51–53]. Moreover, Buckingham et al. (2023) [54] suggested a significative association between digital self-efficacy and mental wellbeing and life satisfaction. Our findings, together with the existing literature, suggest that more training in digital health competences is needed to obtain better outcomes in mental health and well-being, and to reduce existing gaps in the use of technology. Furthermore, participants emphasized that technology should be user-centered and offer real value to users. According to the participants, it should also be supported by scientific evidence and implemented through a clear implementation strategy. This challenge has been previously reported, noting that despite the availability of many mobile apps for mood disorders and mental health, few are supported by scientific evidence regarding their effectiveness and usability [55].

Finally, our study addressed the potential risks and adverse effects of digital tools in depression care. Professionals expressed concerns about unmoderated forums or support spaces on social networks where people with depression communicate with each other, fearing that negative reinforcement could worsen symptoms. While such platforms can foster emotional expression and reduce stigma (especially when anonymity is allowed) [52] they are safer when moderated by mental health experts or trained professionals [53]. However, professionals in our study were reluctant to assume this supervisory role. Both, the potential risks-adverse effects and the promotion of the use of safe, evidence-based digital health tools that are user-centered represent a challenge that needs to be addressed.

### Strengths and limitations

One of the main strengths of this study is that, despite the small size of the sample in some sessions, it has managed to include a wide range of participants, both in terms of people diagnosed with depression and different professional profiles. The sample varied in age, gender, comorbidities, and digital literacy levels. The workshops were prepared and facilitated by a design thinking expert unaffiliated with the research team, minimizing potential bias. In addition, the study highlights the importance of stakeholder involvement through empathy-based processes before developing or implementing digital interventions.

However, several limitations should be considered. Recruiting people diagnosed with depression proved challenging, and many who initially expressed interest did not attend. Nevertheless, data saturation was achieved. Another limitation is that the sessions were not audio-recorded due to the format of the workshops. Although written materials captured the main insights, the absence of recording may have resulted in the loss of some relevant contributions to the dynamics, as well as conversations among participants.

## Conclusion

Digital health holds significant potential to improve the daily lives of people with depressive symptoms. To realize this potential, it is essential to understand user needs firsthand and incorporate the perspectives of all stakeholders in the design and development processes. Ongoing challenges include ensuring that digital tools are evidence-based, safe, effective and user-centered, with clearly defined strategies for implementation and evaluation.

## Supporting information

S1 Data Raw data from the empathy workshops. (PDF)
