# Supplementary material for: Stakeholder perspectives on depression management: A design thinking exploration for person-centered digital health
Source: PLoS One. 2026 Feb 9;21(2):e0341431. doi: 10.1371/journal.pone.0341431 (PMC12885253; doi:10.1371/journal.pone.0341431)

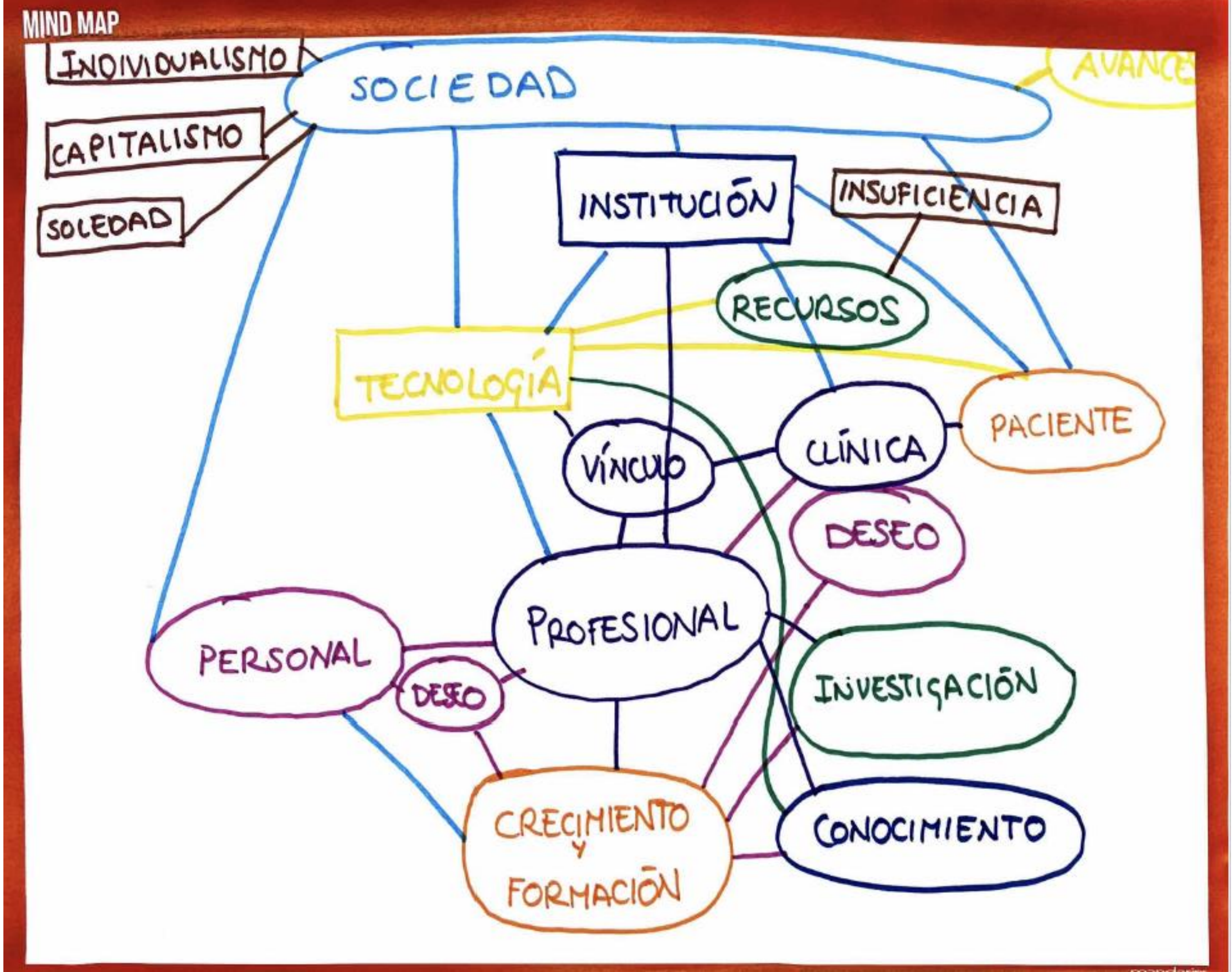
MIND MAP
INDIVIDUALISMO
SOCIEDAD
AVANCE
CAPITALISMO
SOLEDAD
INSTITUCIÓN
INSUFICIENCIA
RECURSOS
TECNOLOGÍA
VÍNCULO
CLÍNICA
PACIENTE
DESEO
PROFESIONAL
PERSONAL
DESEO
INVESTIGACIÓN
CRECIMIENTO Y FORMACIÓN
CONOCIMIENTO

EL VIAJE

ENERO | FEBRERO | MARZO | ABRIL | MAYO | JUNIO | JULIO | AGOSTO | SEPTIEMBRE | OCTUBRE | NOVIEMBRE | DICIEMBRE

Carnavales
y
semana santa
=
fiestas

bajón
después
fiestas

valoración de
los propósitos

periodo
sin
cole
(vacacional) ☺
BUEN TIEMPO

text

actividad aire libre

invierno
· MAL TIEMPO · ☹

propósitos de año nuevo

post
navidad +
cuesta enero

DPP educación
emocional,
entender fases
problema, no sólo inmediatez.
Es un proceso

escucha
conversacional

inicio
rutinas

mandarina newton

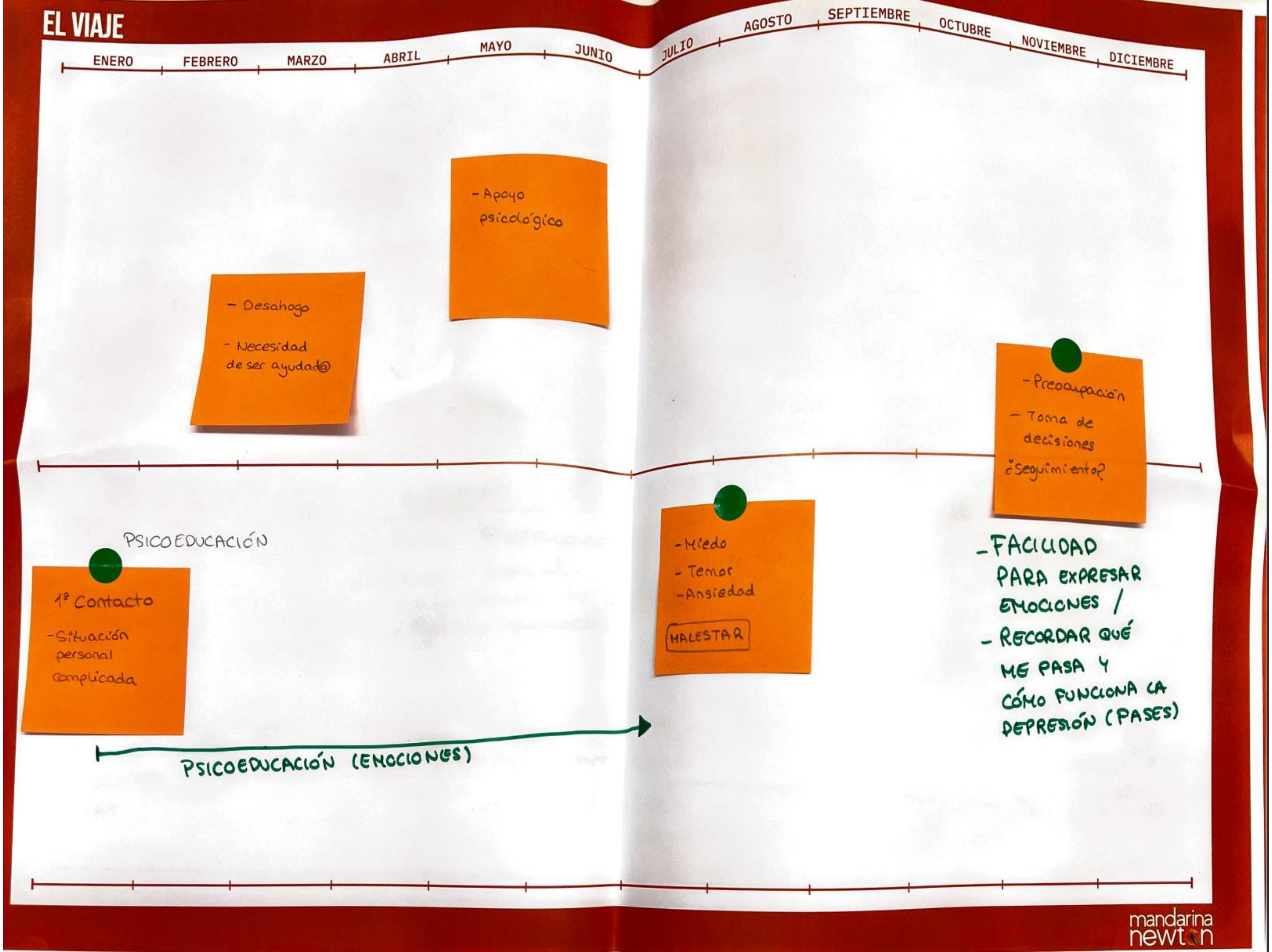
EL VIAJE
ENERO
FEBRERO
MARZO
ABRIL
MAYO
JUNIO
JULIO
AGOSTO
SEPTIEMBRE
OCTUBRE
NOVIEMBRE
DICIEMBRE
- Desahogo
- Necesidad de ser ayudad@
- Apoyo psicológico
- Preocupación
- Toma de decisiones
¿Seguimiento?
PSICOEDUCACIÓN
1º Contacto
- Situación personal complicada
- Miedo
- Temor
- Angiedad
MALESTAR
- FACILIDAD PARA EXPRESAR EMOCIONES /
- RECORDAR QUÉ ME PASA Y CÓMO FUNCIONA LA DEPRESIÓN (PASES)
PSICOEDUCACIÓN (EMOCIONES)
mandarina newton

MIND MAP

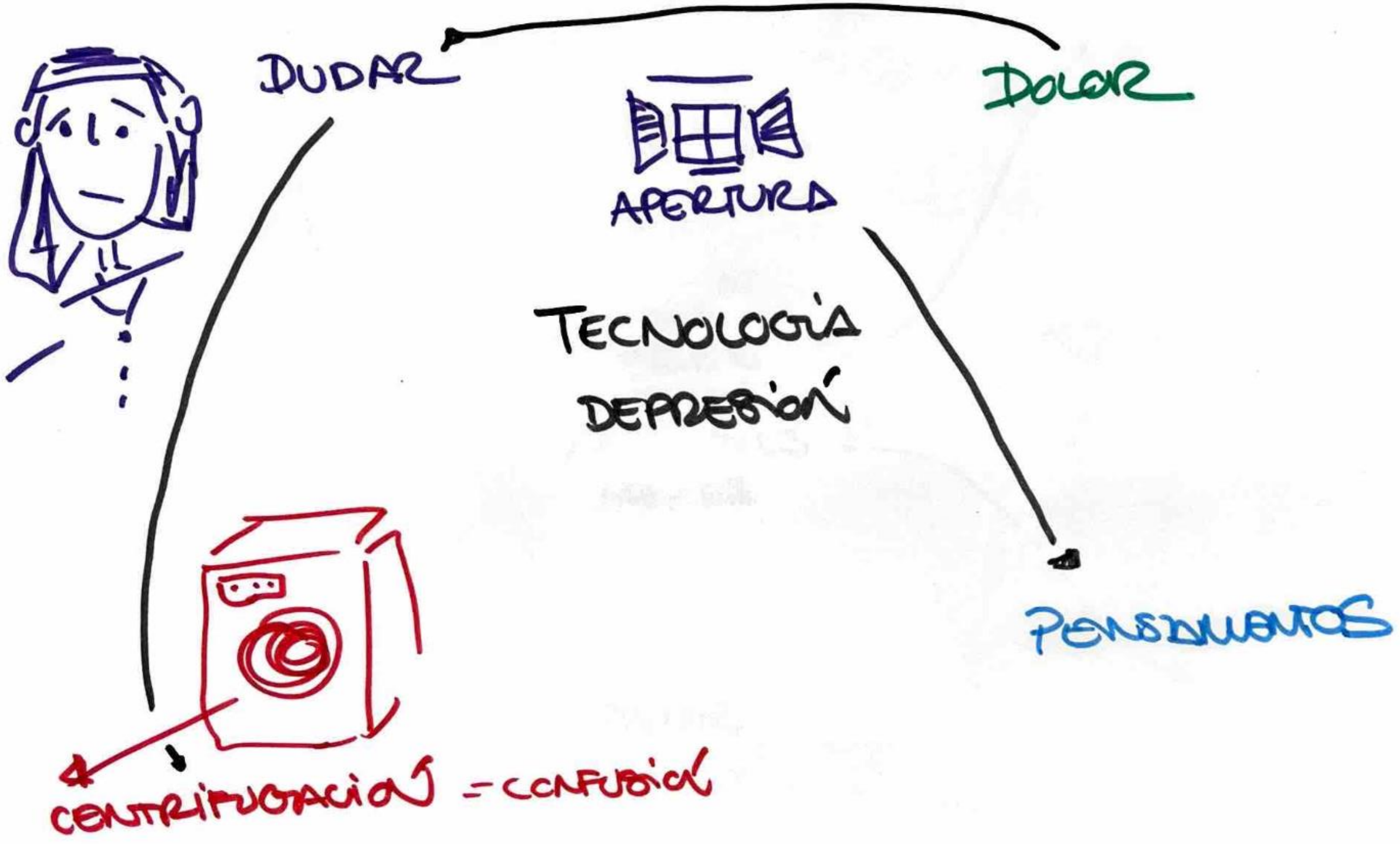

mandarina newton

STORYBOARD

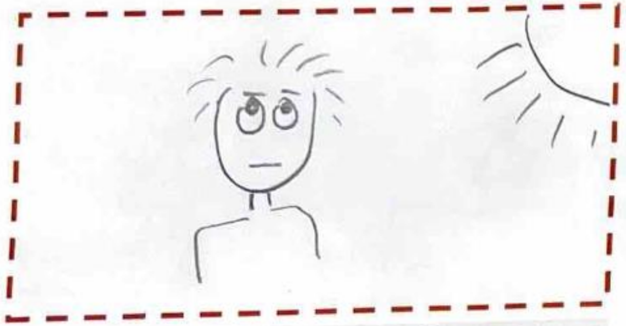

1. LLEVO DIAS QUE ME SIENTO MAL. NECESITO AYUDA.

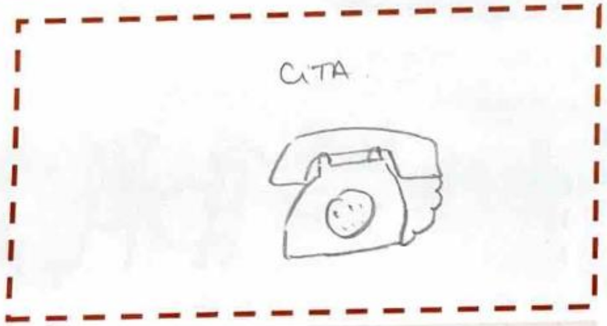

2. LLAMO CENTRO / APLIC MI CITA PREV.

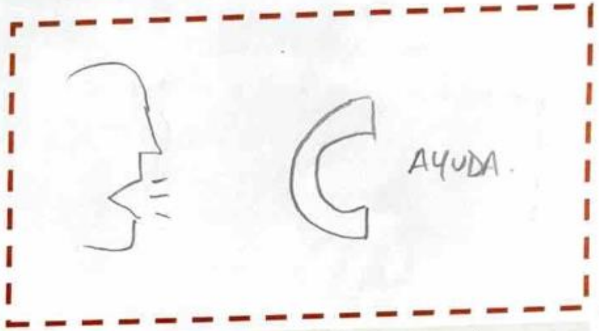

3. Escucho su problema.
- AYUDA.
- COMENTO CON MEDICO.

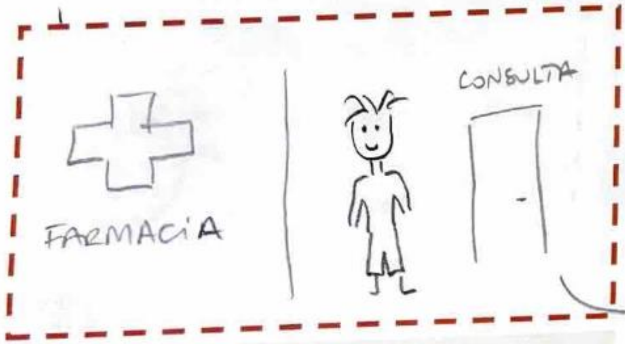

4.
- TRATAMIENTO. Y CITAR para ver evolución
- Genera consulta presencial

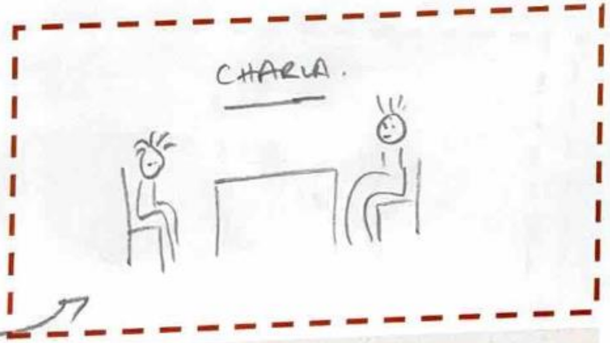

5.
- ESCUCHA ACTIVA.
- ANIMAR Y OFRECER CONSUELO.
- NORMALIZAR LO QUE TANTO LE ANGUSTIA

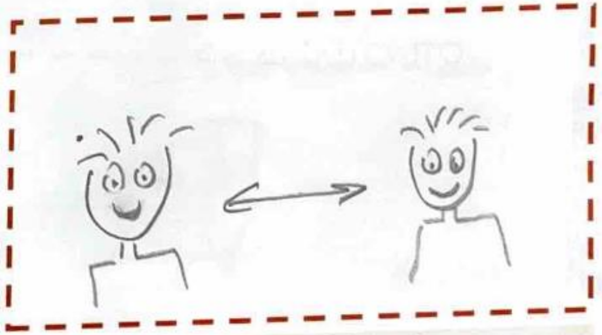

6. SEGUIMIENTO
- Presencial
- Telefónico

OFRECERLES APLICACIÓN.

CONTINUARÁ

STORYBOARD

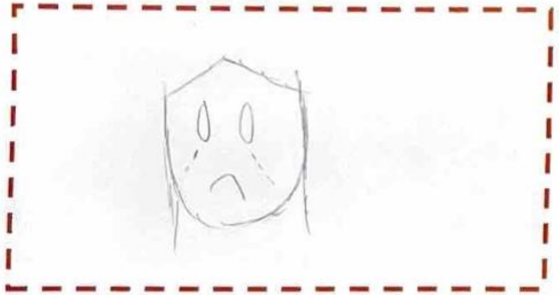

1.

Situación complicada (depresión / ansiedad)

- Conocer la APP

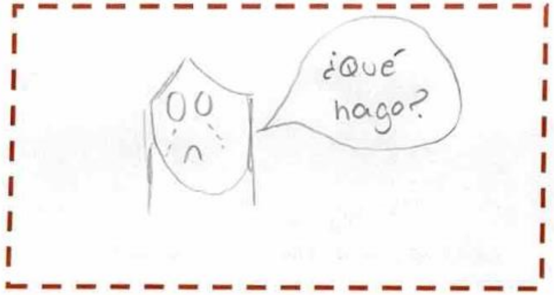

2.

Toma la decisión de pedir ayuda →

Recurrir a la app

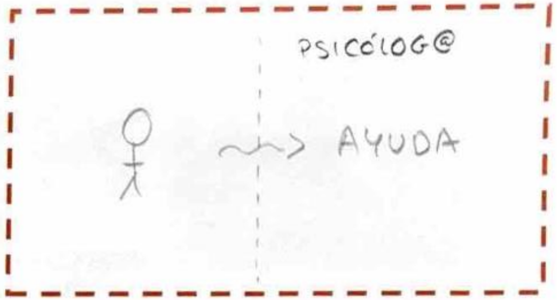

3.

- Atención psicológica
- Psicoeducación

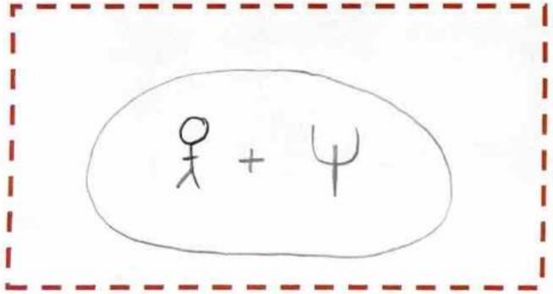

4.

VÍNCULO CON EL / LA PROFESIONAL

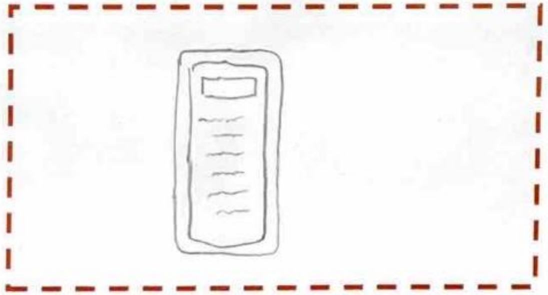

5.

Ayuda en la toma de decisiones en la APP (registro)

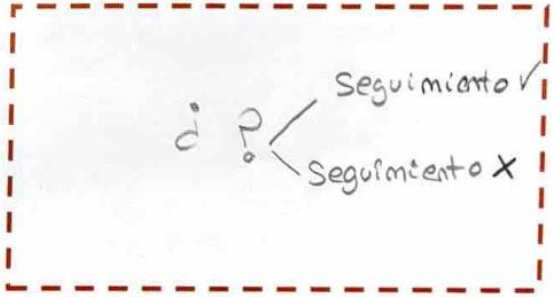

6.

mandarina newton

MIND MAP

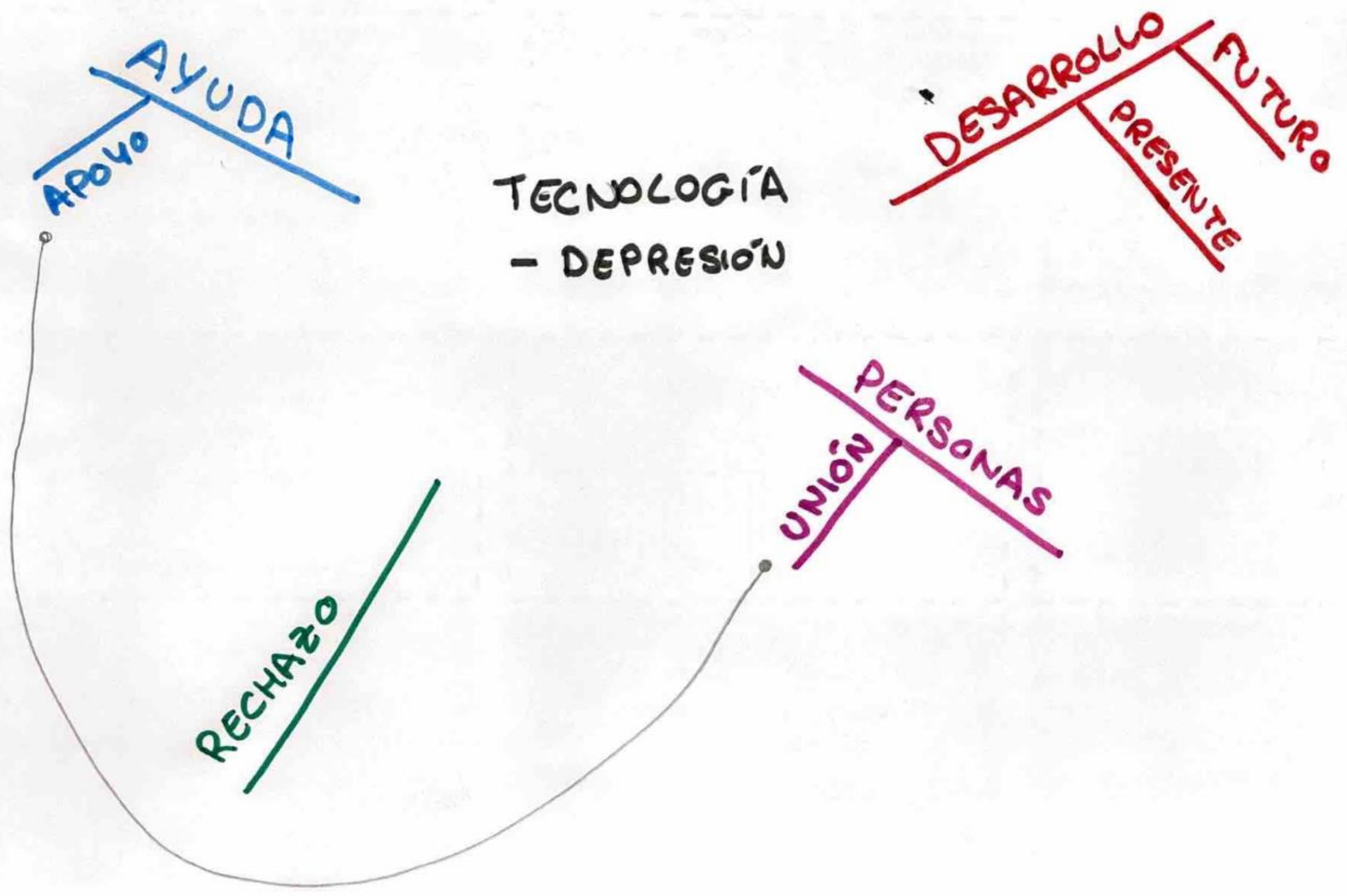
AYUDA
APOYO
TECNOLOGÍA
- DEPRESIÓN
DESARROLLO
PRESENTE
FUTURO
PERSONAS
UNIÓN
RECHAZO

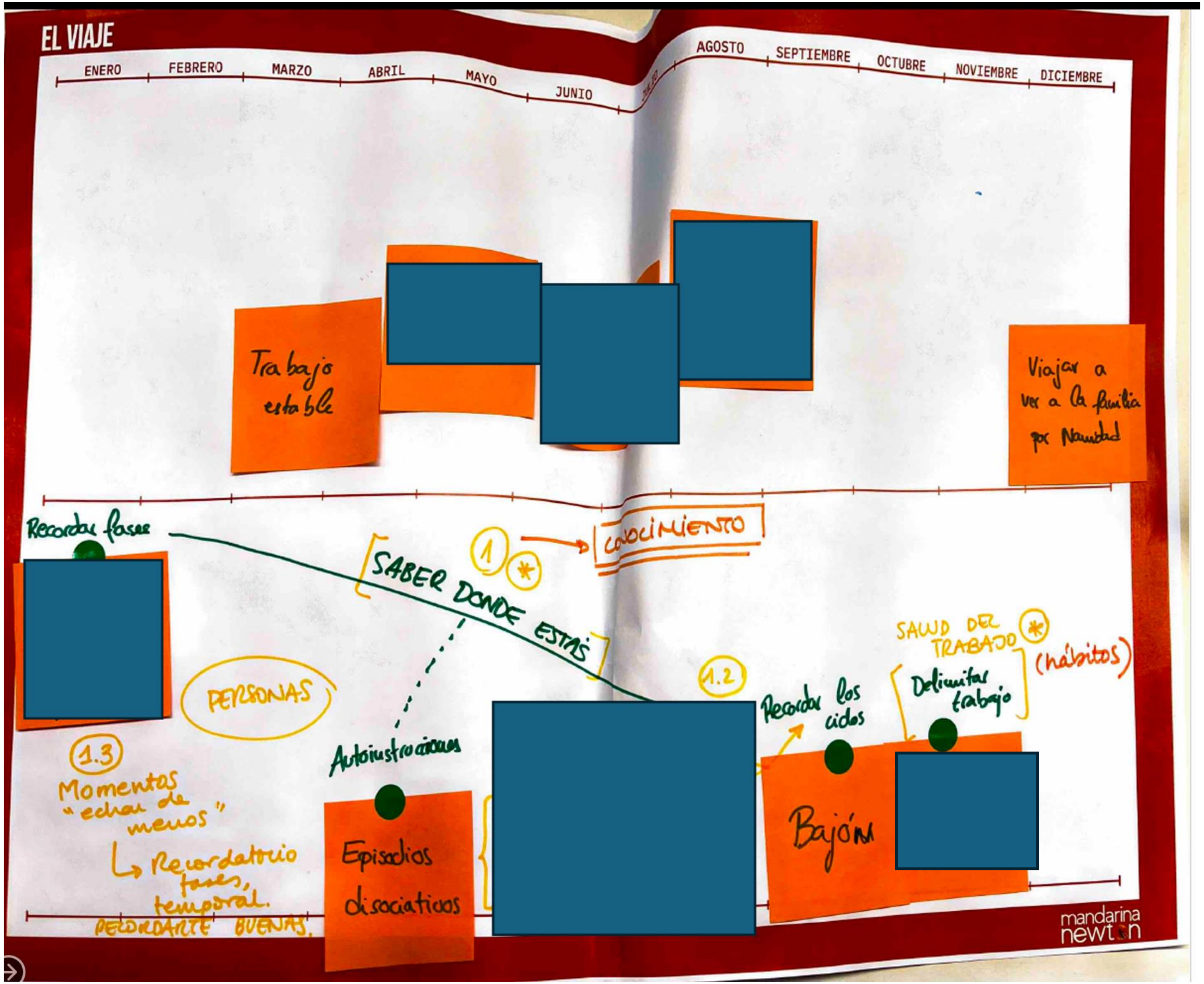
EL VIAJE
ENERO
FEBRERO
MARZO
ABRIL
MAYO
JUNIO
JULIO
AGOSTO
SEPTIEMBRE
OCTUBRE
NOVIEMBRE
DICIEMBRE
Trabajo estable
Viajar a ver a la familia por Navidad
Recordar fases
SABER DONDE ESTÁS
1
*
CONOCIMIENTO
PERSONAS
1.3
Momentos "echar de menos"
↳ Recordatorio fases, temporal.
RECORDARTE BUENAS
Autoinstrucciones
Episodios disociativos
1.2
Recordar los ciclos
Bajón
SALUD DEL TRABAJO
*
(hábitos)
Delimitar trabajo
mandarina newton

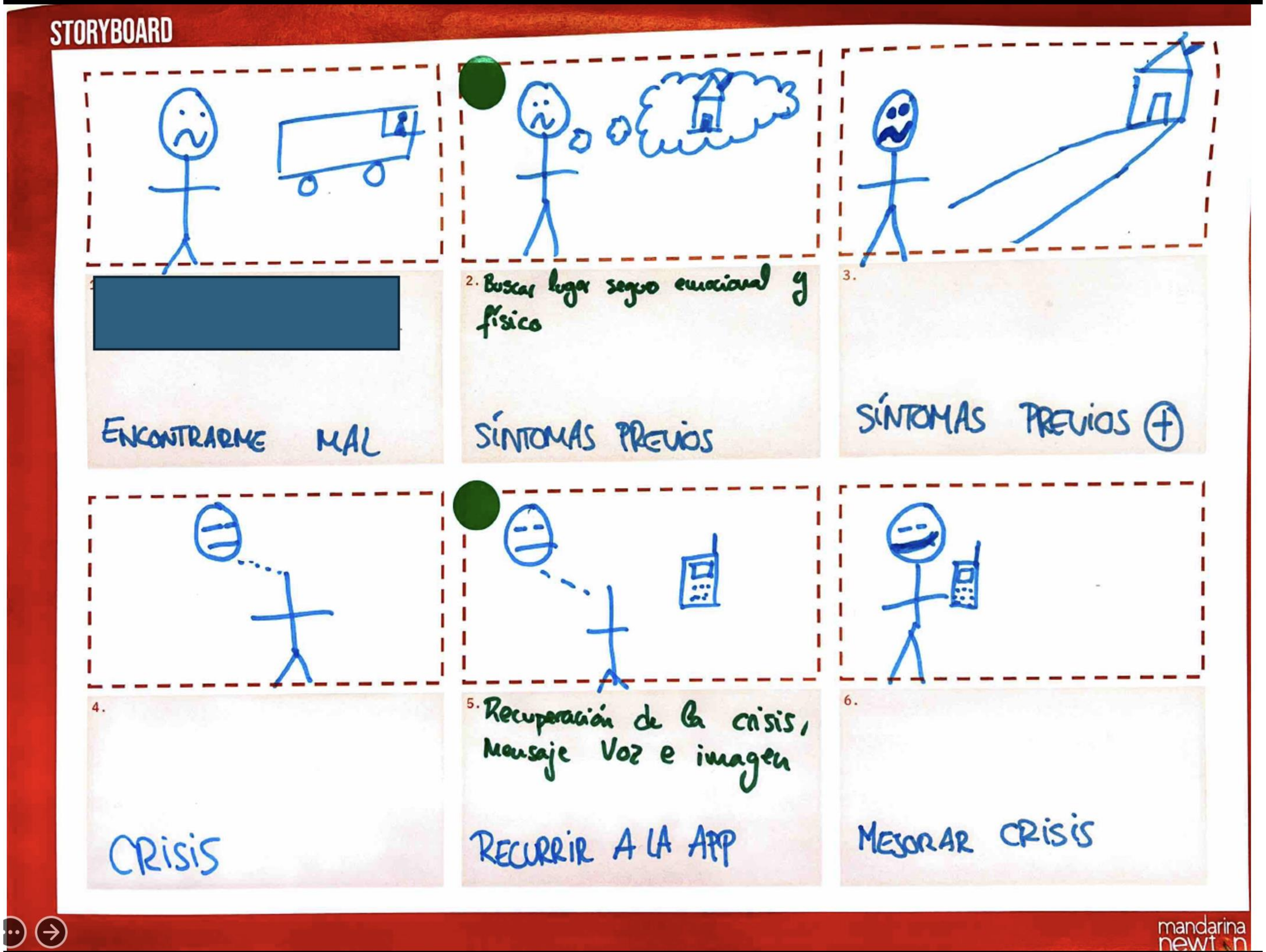
STORYBOARD
[redacted]
ENCONTRARME MAL
2. Buscar lugar seguro emocional y físico
SÍNTOMAS PREVIOS
3.
SÍNTOMAS PREVIOS +
4.
CRISIS
5. Recuperación de la crisis, Mensaje Voz e imagen
RECURRIR A LA APP
6.
MEJORAR CRISIS
mandarina newton

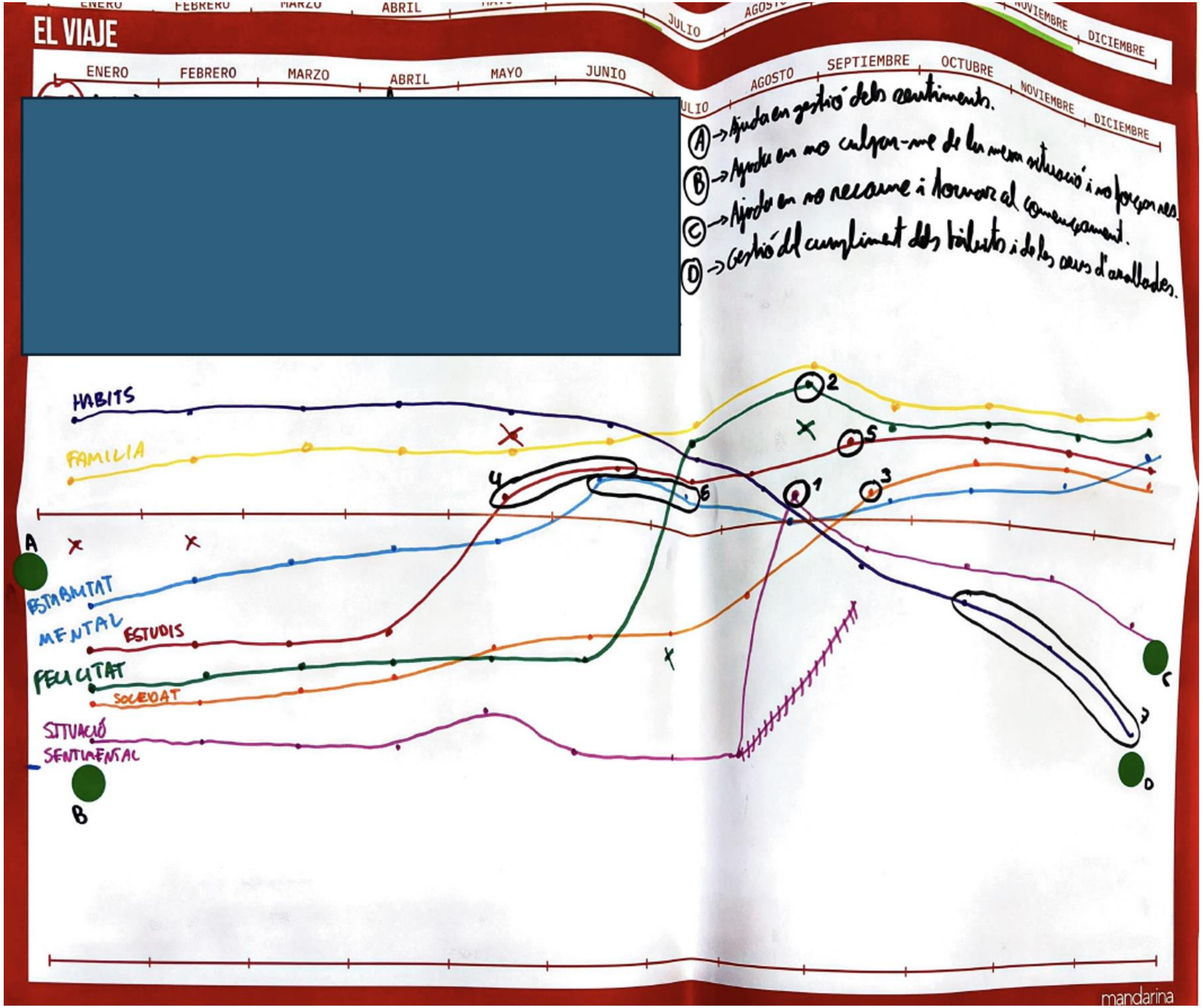
EL VIAJE
ENERO
FEBRERO
MARZO
ABRIL
MAYO
JUNIO
JULIO
AGOSTO
SEPTIEMBRE
OCTUBRE
NOVIEMBRE
DICIEMBRE
(A) → Ajuda en gestió dels sentiments.
(B) → Ajuda en no culpar-me de la meva situació i no forçarnes.
(C) → Ajuda en no recaure i tornar al començament.
(D) → Gestió del compliment dels tractaments i de les [illegible]
HABITS
FAMILIA
ESTABILITAT MENTAL
ESTUDIS
FELICITAT
SOCIETAT
SITUACIÓ SENTIMENTAL
A
B
C
D
mandarina

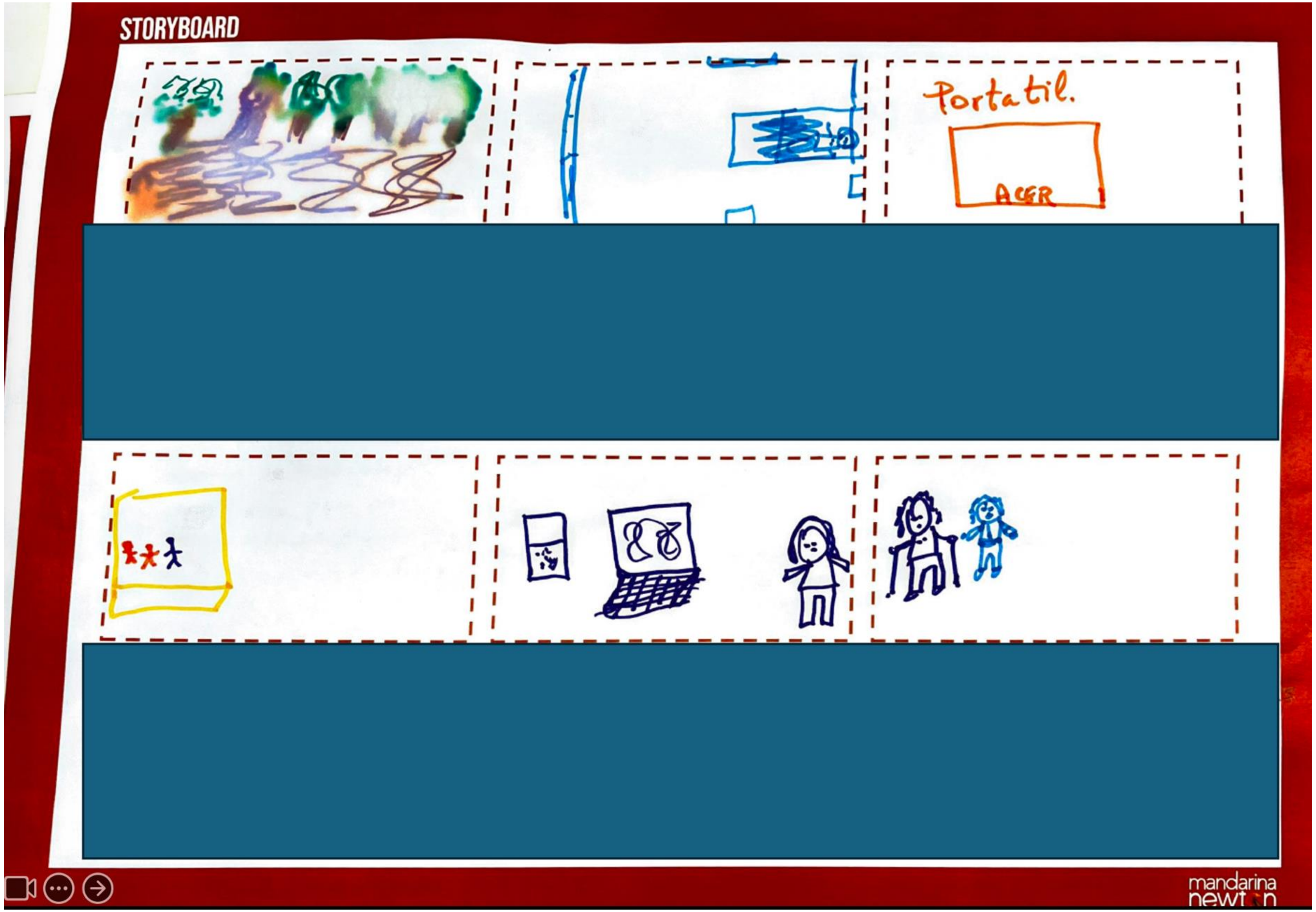
STORYBOARD
Portatil.
ACER
mandarina newton

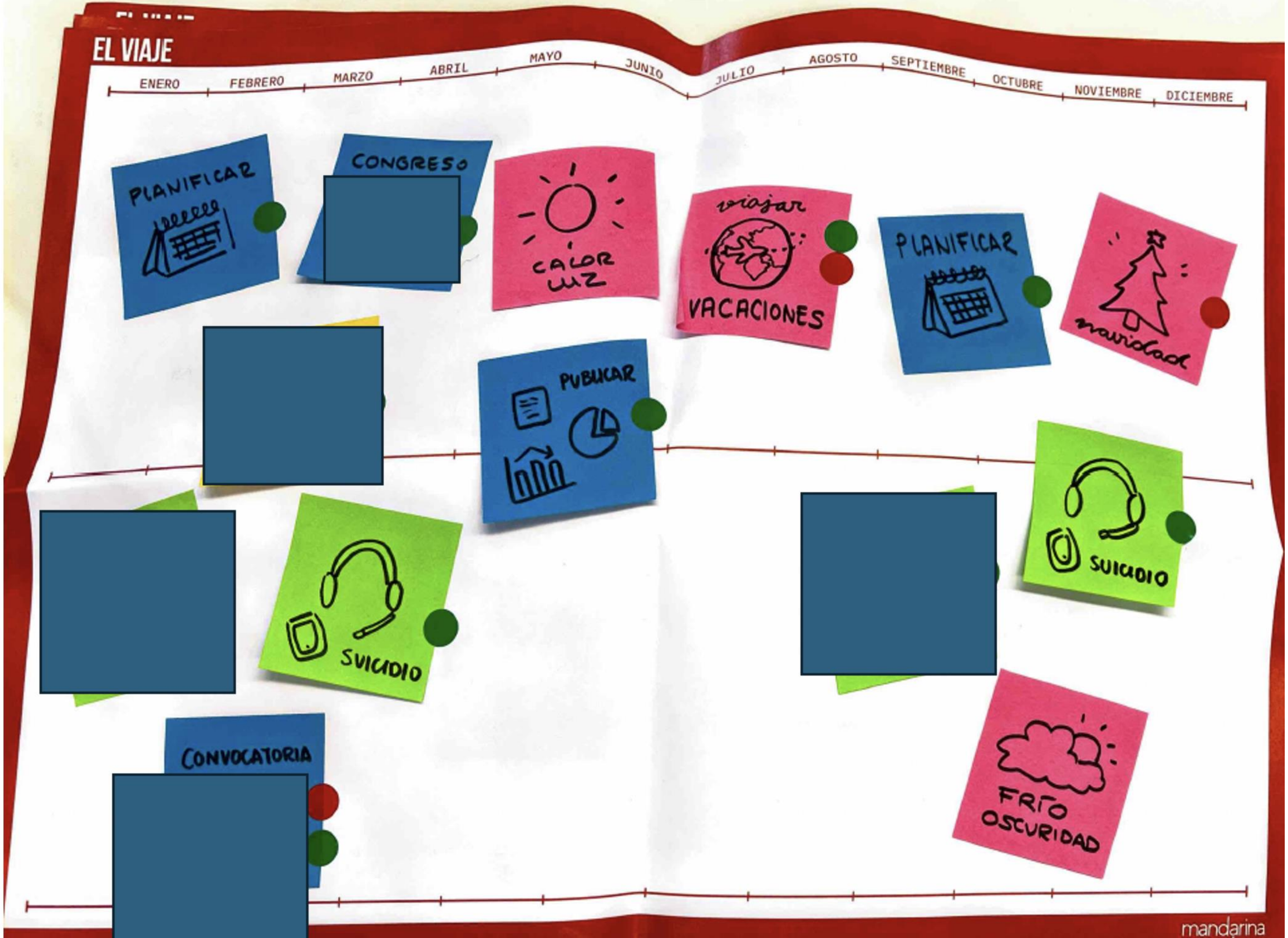
EL VIAJE
ENERO
FEBRERO
MARZO
ABRIL
MAYO
JUNIO
JULIO
AGOSTO
SEPTIEMBRE
OCTUBRE
NOVIEMBRE
DICIEMBRE
PLANIFICAR
CONGRESO
CALOR
LUZ
viajar
VACACIONES
PLANIFICAR
navidad
PUBLICAR
SUICIDIO
SUICIDIO
CONVOCATORIA
FRIO
OSCURIDAD
mandarina

STORYBOARD

BASAL

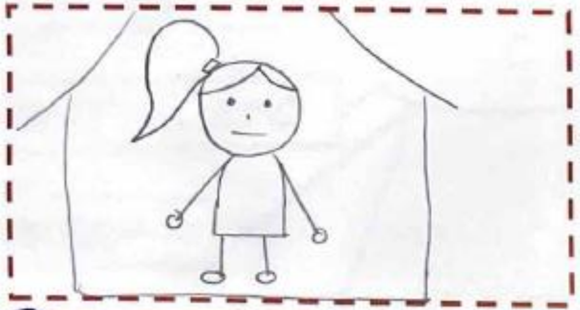

1. Organización de horarios
   - sueño
   - medicación
   - actividades (abvd)
   - ejercicio / social / ocupacional

   * Crear junto al terapeuta.

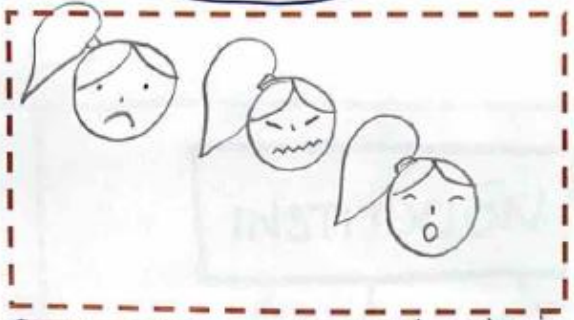

2. Registro emocional diario
   - -> crear línea base y contextualiz.
   - -> estimular afrontamiento adaptativo
     - • propuesta de actividad
     - • búsqueda apoyo social

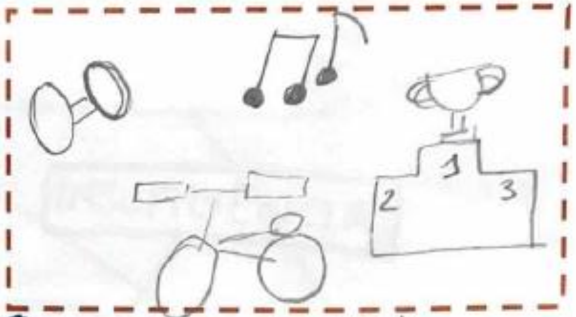

3. Registro actividad
   - -> crear línea base
   - -> reforzar avances ~~(puntos)~~
   - -> puntualizar aspectos positivos.
   - -> feedback (puntos, records, ...)

SITUACIONES DE CRISIS / DIFICULTAD

4. Intervención en situaciones de ansiedad
   - ejercicios de relajación por ej.
   - acompañamiento por bot
     - • tips, aceptación, etc.

5. Intervención en desajustes de sueño y otras alteraciones.
   - medidas de higiene de sueño
   - ejercicios relajación

6. Situaciones de crisis grave y de riesgo autolítico.
   - contactos de emergencia
   - búsqueda / aviso a figuras de referencia.
   - contacto con profesional.

mandarina newton

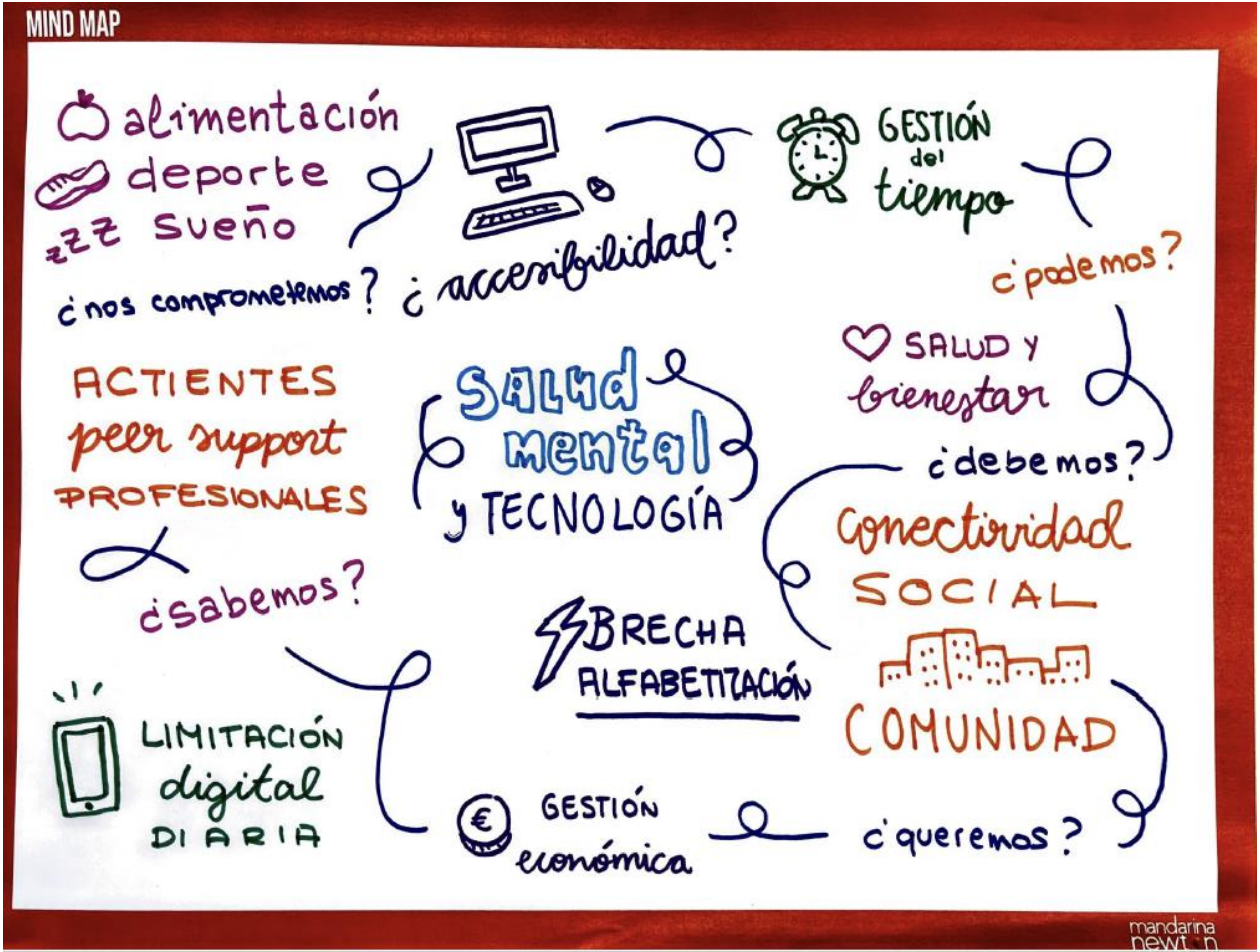
MIND MAP
alimentación
deporte
zZZ sueño
¿nos comprometemos?
¿accesibilidad?
GESTIÓN del tiempo
¿podemos?
SALUD Y bienestar
ACTIENTES
peer support
PROFESIONALES
Salud mental y TECNOLOGÍA
¿debemos?
Conectividad SOCIAL
¿sabemos?
BRECHA ALFABETIZACIÓN
COMUNIDAD
LIMITACIÓN digital DIARIA
GESTIÓN económica
¿queremos?
mandarina newton

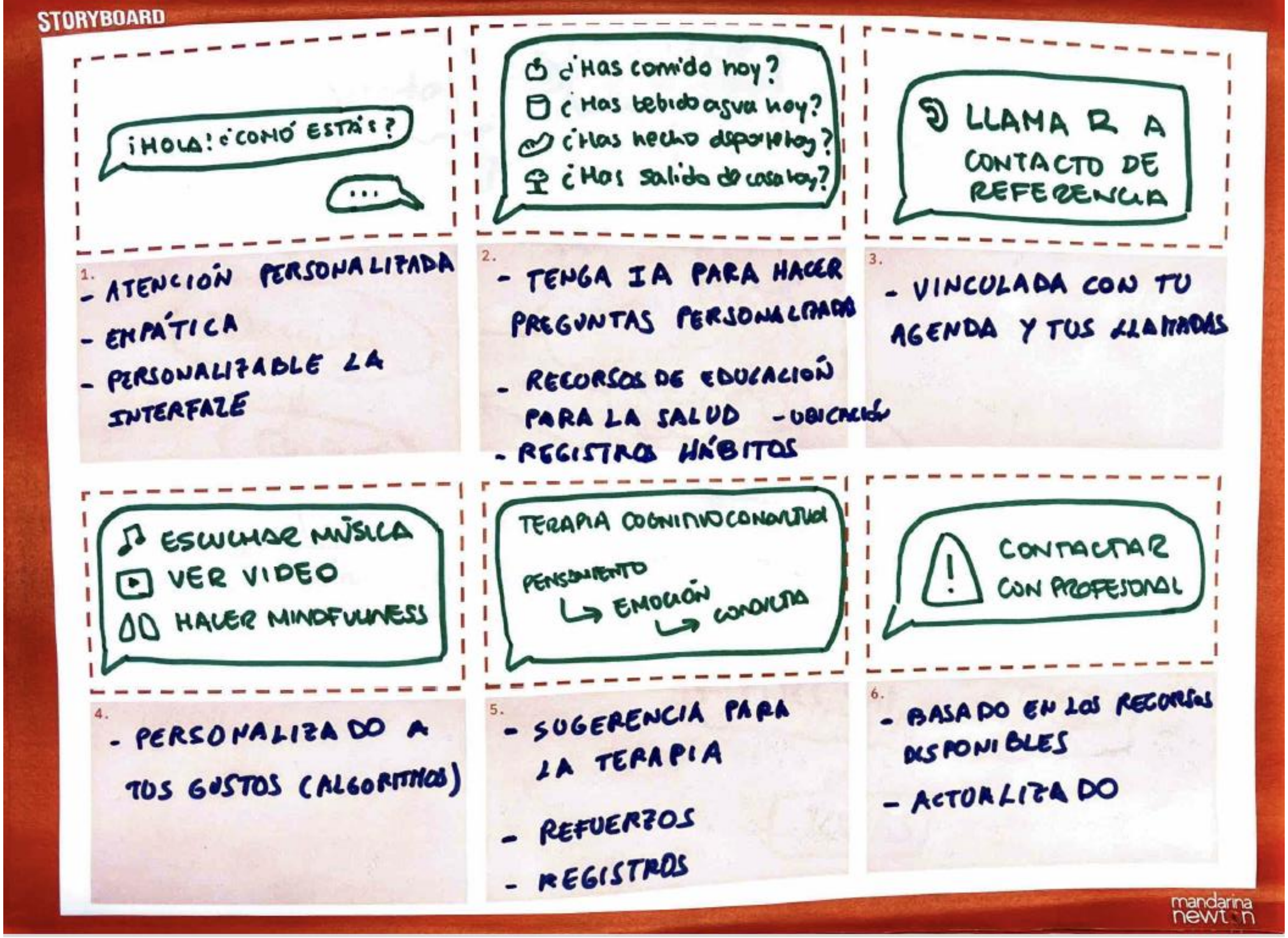
STORYBOARD
¡HOLA! ¿CÓMO ESTÁS?
...
¿Has comido hoy?
¿Has bebido agua hoy?
¿Has hecho deporte hoy?
¿Has salido de casa hoy?
LLAMAR A CONTACTO DE REFERENCIA
1.
- ATENCIÓN PERSONALIZADA
- EMPÁTICA
- PERSONALIZABLE LA INTERFAZE
2.
- TENGA IA PARA HACER PREGUNTAS PERSONALIZADAS
- RECURSOS DE EDUCACIÓN PARA LA SALUD
- UBICACIÓN
- REGISTRAR HÁBITOS
3.
- VINCULADA CON TU AGENDA Y TUS LLAMADAS
ESCUCHAR MÚSICA
VER VIDEO
HACER MINDFULNESS
TERAPIA COGNITIVO CONDUCTUAL
PENSAMIENTO
EMOCIÓN
CONDUCTA
CONTACTAR CON PROFESIONAL
4.
- PERSONALIZADO A TUS GUSTOS (ALGORITMOS)
5.
- SUGERENCIA PARA LA TERAPIA
- REFUERZOS
- REGISTROS
6.
- BASADO EN LOS RECURSOS DISPONIBLES
- ACTUALIZADO
mandarina newton

TALLER EMPATÍA

PACIENTES CANARIAS

Aislamiento — ojo! con desconectar

Soledad — - POR - Inseguretat

Tristeza —

- Falta de energía — - Vellesa

cansament — - Futur — - Salut

* Familia / Amics — - t*

Economia — parella

[Caos mundo — valors personals

[Política

Preguntes existencials — tener raón

→ Estabilitat mental

TALLER EMPATÍA

PACIENTES MÁLAGA

Apps.
- CULPA.
- RECAURE
- TORNAR.
- GESTIÓ SENTIMENTS/EMOCIONS

Hàbits || situació sentimental

③ Comorbilitat → info
personal/familiar

④ Meditació / Deporte.
Por a l'enganche?

⑤ Rutines

⑥ FOTOS - RECORS

⑦ Comunicación
⑧ Personalitzable que puedas llenarla de recursos.
⑨ Dormir
- cuentos
⑩ Referentes

①
VOZ
PULSERA
ANSIEDAD → DEPRE
- hablar
- informar pasos...

② DIARIO → compatible
+ ex. + anàlisi amb el equip psico.

TALLER EMPATIA

PROFESIONALES
MÁLAGA

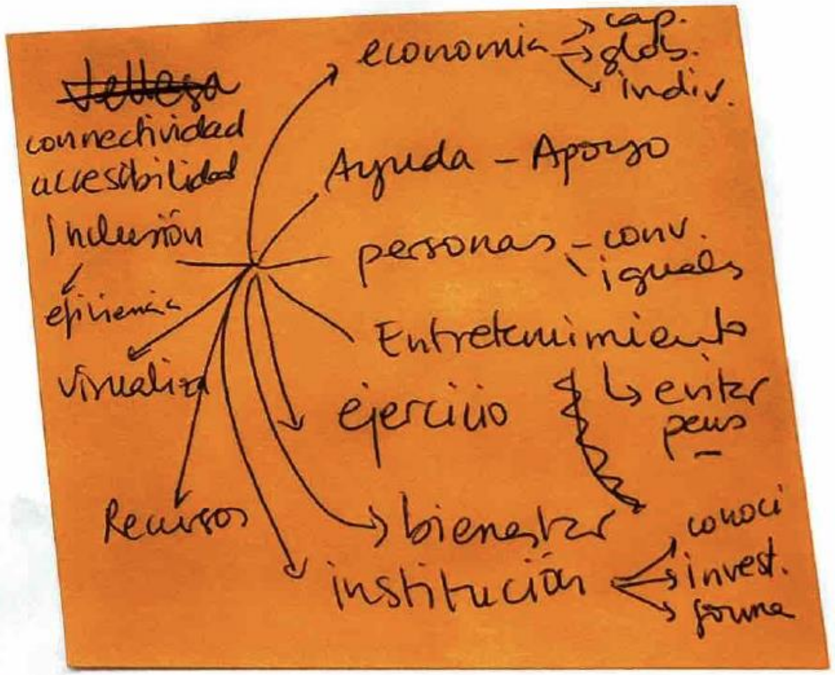

Supplement: S1 Data — (PDF) [file pone.0341431.s001.pdf]
